# Effects of Systemic Vibratory Therapy Combined with a Physical Activity Program in Older Adults on Fall Risk, Balance, Physical Conditioning, and Neuromuscular Variables: Study Protocol for a Randomized Controlled Trial

**DOI:** 10.3390/healthcare14121723

**Published:** 2026-06-15

**Authors:** Alexandre Gonçalves de Meirelles, Ygor Teixeira da Silva, Julio Cesar de Oliveira Muniz Cunha, Luis Leitão, Leandro Alberto Calazans Nogueira, José Vilaça-Alves, Mário Bernardo Filho, Igor Ramathur Telles de Jesus, Estêvão Rios Monteiro

**Affiliations:** 1 Graduate Program in Rehabilitation Science, Centro Universitário Augusto Motta (PPGCR/UNISUAM), Rio de Janeiro 21032-060, Brazil; alexandremeirelles@souunisuam.com.br (A.G.d.M.); ygor.silva@souunisuam.com.br (Y.T.d.S.); juliomuniz@souunisuam.com.br (J.C.d.O.M.C.); leandronogueira@souunisuam.com.br (L.A.C.N.); ijesus@souunisuam.com.br (I.R.T.d.J.); 2 Instituto Politécnico de Setúbal, Escola Superior de Educação, CIEQV, 2910-761 Setúbal, Portugal; luis.leitao@ese.ips.pt; 3 Life Quality Research Centre (CIEQV), 2910-761 Setúbal, Portugal; 4 Research Centre in Sports, Health and Human Development (CIDESD), 6201-001 Covilhã, Portugal; 5 Physiotherapy Department, Federal Institute of Rio de Janeiro (IFRJ), Rio de Janeiro 21710-240, Brazil; 6 Department of Sports Sciences, Exercise and Health, Universidade de Trás-os-Montes e Alto Douro, 5000-801 Vila Real, Portugal; josevilaca@utad.pt; 7 Research Center in Sports Sciences, Health Sciences and Human Development (CIDESD), 5001-801 Vila Real, Portugal; 8 Laboratório de Vibrações Mecânicas e Práticas Integrativas, Instituto de Biologia Roberto Alcantara Gomes, Universidade do Estado do Rio de Janeiro, Rio de Janeiro 20950-003, Brazil; bernardofilhom@gmail.com

**Keywords:** fall risk prevention, older adults, physiotherapy in older adults, physical activities in older adult, whole-body vibration training

## Abstract

**Introduction**: Population aging is a growing and challenging phenomenon, primarily due to its association with functional decline and sarcopenia, which increase the risk of falls. These events have significant impacts on public health and the quality of life of older adults. Regular physical activity has shown benefits in reducing falls and their consequences, with systemic vibratory therapy (SVT) emerging as a promising strategy to mitigate these adverse outcomes. However, evidence on the actual effectiveness of this therapeutic approach remains limited, as does clarity regarding optimal body position, protocol parameters, and equipment when combined with physical activity programs. **Objectives**: To compare the effect of systemic vibratory therapy (SVT) associated with a physical activity program on the perception of fear of falling in older adults (M01.060.116.100). As secondary outcomes, the study will assess functional physical conditioning, electromyographic activity, muscular synergy, and center of pressure oscillation in this population. **Methods**: A randomized controlled clinical trial with blinded outcome assessors and blinded statistical analysis will be conducted with 192 older adults participating in the UNATI/UNISUAM program. Participants will be allocated into three groups: (A) usual physical activity; (B) usual physical activity + SVT in a semi-squat position; and (C) usual physical activity + SVT in a seated position. Assessments will include sociodemographic data, concern about falling assessed using the Falls Efficacy Scale-International (FES-I), physical performance (2 min stationary march test), surface electromyography of the tibialis anterior and medial gastrocnemius muscles, along with posturography using a force platform. **Results**: This study will provide information on outcomes related to fall risk, balance, physical fitness, and neuromuscular variables in older adults undergoing two distinct SVT protocols. **Clinical Trials Registration**: Brazilian Registry of Clinical Trials RBR-68pry5j. Registered on 8 December 2025.

## 1. Introduction

Population aging is a growing global phenomenon, especially in developing countries such as Brazil [1]. According to the World Health Organization (WHO) [1], individuals aged 60 or over are considered elderly in countries like Brazil. Projections from the Brazilian Institute of Geography and Statistics (IBGE) indicate a significant increase in the proportion of elderly individuals by 2050 [2,3].

As aging progresses, several physiological changes become evident, including loss of muscle mass and strength, reduced reflexes and motor reaction time, sensory and cognitive decline, as well as hormonal and metabolic alterations [4]. These factors contribute to an increased risk of falls, which place a substantial burden on the healthcare system, including prolonged hospitalizations, loss of autonomy, and reduced quality of life among older adults [5,6]. The WHO estimates that around 37.3 million severe falls occur globally each year, resulting in approximately 684,000 deaths (https://www.who.int/news-room/fact-sheets/detail/falls. Accessed on 31 December 2025). In Brazil alone, over 1.7 million hospitalizations due to falls among the elderly were recorded between 2000 and 2020, particularly among women aged over 80 years in the Southeast region [5]. Fractures were the main complications, mostly affecting the lower limbs, followed by upper limbs and the spine, with 60% of falls occurring at home [7].

In this context, promoting physical activity among older adults is essential. Physical activity provides numerous benefits, including reduced muscle loss, improved autonomy, increased social interaction, enhanced functional independence, and reduced risk of age-related diseases [8,9,10]. Moreover, it serves as an effective non-pharmacological strategy for fall prevention. Physical activity refers to any bodily movement that results in energy expenditure, while physical exercise is a planned and structured activity [9]. In this regard, the WHO advocates for the concept of active aging, which emphasizes the promotion of autonomy, functional independence, social participation, and healthy life expectancy [11].

A promising intervention is systemic vibratory therapy (SVT), which uses vibration platforms to provide mechanical stimuli capable of eliciting muscular, proprioceptive, and hormonal responses [12,13]. Reduced muscle strength, impaired sensory integration, and altered postural adjustments in older adults are associated with an increased risk of falls [11]. Previous studies suggest that SVT may improve lower limb muscle performance, balance, mobility, and physical function in older populations, although the magnitude of these effects appears to depend on protocol characteristics such as frequency (F), peak-to-peak displacement (D), exposure time, rest intervals, and body position during vibration exposure [13,14]. Despite these potential benefits, the ideal SVT protocol for older adults is not yet defined. Current evidence still shows considerable heterogeneity regarding vibration parameters, intervention duration, equipment characteristics, and participant positioning, which limits the establishment of standardized recommendations for clinical practice [12]. Body position during SVT can be particularly relevant, as it can modify axial load, lower limb muscle activation, postural demand, comfort, and safety. The semi-squat position can increase active lower limb engagement and proprioceptive stimulation, while the seated position can reduce weight-bearing demands and provide a more tolerable alternative for frail, sedentary, or low-functional older adults [13,14,15]. Therefore, comparing these positions can help clarify whether the effects of SVT are primarily related to exposure to mechanical vibration itself or to the combination of vibration with active postural and muscular demands.

Beyond objective fall risk metrics, concern about falling is a patient-centered outcome that can constrain activity participation and undermine adherence to exercise-based prevention strategies. Conceptually, falls efficacy reflects an older adult’s perceived capability to prevent and manage falls in daily life; thus, quantifying change in this construct provides clinically relevant information that complements performance- and physiology-based outcomes [16,17]. The Falls Efficacy Scale-International (FES-I) is among the most widely used instruments to assess concerns about falling, and recent syntheses support its measurement properties and interpretability for older populations, although responsiveness benchmarks may vary across settings [18]. Accordingly, the present trial defines change in FES-I from baseline to post-intervention as the primary endpoint, while physical conditioning, neuromuscular activity, and postural control outcomes are retained as secondary measures to help contextualize potential physiological pathways of systemic vibratory therapy combined with habitual physical activity. Thus, this study aims to compare the effects of SVT associated with a physical activity program on fall concern in older adults. As secondary outcomes, the study will assess functional physical fitness, electromyographic activity, muscle synergy, and center of pressure oscillation [12,13,14,15].

Our hypothesis is that SVT combined with habitual physical activity will produce greater improvements in concern about falling, functional fitness, postural control, and neuromuscular variables compared with habitual physical activity alone. We also hypothesize that the semi-squat and seated SVT positions may produce different neuromuscular responses due to differences in axial load, lower limb muscle activation, and postural demand.

## 2. Materials and Methods

### 2.1. Study Design

This study is a randomized controlled clinical trial with blinded outcome assessors and blinded statistical analysis. Due to the perceptible nature of the vibration stimulus and the absence of a sham-vibration condition, participant blinding cannot be fully guaranteed. Therefore, blinding procedures will focus on maintaining the outcome assessors and the researchers responsible for statistical analysis unaware of group allocation throughout the trial. The study will adhere to the recommendations of the CONSORT 2025 statement [19]. The primary aim is to investigate the effects of SVT combined with a physical activity program on the concern about falling, functional physical conditioning, and neuromuscular variables in older adults.

A total of 192 older adults enrolled in the Open University for the Elderly (UNATI) at Centro Universitário Augusto Motta (UNISUAM), Bonsucesso Unit, will be recruited. Participants will be allocated in a 1:1:1 ratio into three groups using sequentially numbered, opaque, sealed envelopes prepared from a computer-generated randomization list. The allocation sequence will be implemented after completion of baseline assessments to preserve allocation concealment. In the control group (Group A), participants will be considered a usual physical activity control group. Participants allocated to this group will continue their regular activities offered by the UNATI/UNISUAM program, including light walking, stretching exercises, activities of daily living, and recreational cognitive activities. No additional vibration stimulus or sham-vibration procedure will be applied. Therefore, the study will compare systemic vibratory therapy combined with habitual physical activity in two distinct positions versus habitual physical activity alone. In the semi-squat intervention group (Group B), participants will engage in the same routine activities and, 30 min after the end of their UNATI sessions, will be directed to the Laboratory of Musculoskeletal Performance of the Graduate Program in Rehabilitation Sciences (PPGCR/UNISUAM), where SVT will be performed in a semi-squat position with knees flexed at 120°. To ensure positioning consistency, the platform base will be pre-marked to standardize foot placement. The intervention will use mechanical vibration at F of 15, 20, and 25 Hz with a D of 5 mm, corresponding to peak accelerations (Apeak) of 2.3 g, 4.1 g, and 6.4 g. Each frequency will be applied for 1 min, with a 1 min rest interval between frequencies, in a cycle repeated three times, totaling 18 min per session. Interventions will occur twice weekly, for a total of 10 sessions. All sessions will be conducted by a research assistant trained in the operation of the vibration platform. Systemic vibratory therapy will be delivered using a Kikos P204IXC alternating oscillatory vibration platform (manufacturer is Kikos) manufactured in São Paulo, Brazil.

The seated intervention group (Group C) will follow the same routine of physical activities and undergo SVT in a seated position on a 30 cm high chair, with knees flexed at 120° and hands resting on the thighs in a comfortable posture, simulating the joint angles of the semi-squat protocol. The vibration parameters will be the same as those used for Group B.

Initial assessments will be conducted before randomization and before the start of the intervention period. Post-intervention assessments will be conducted within 48 h after the last intervention session, preferably at the same time as the initial assessment, in order to minimize possible circadian variations and effects related to fatigue. The same sequence of assessments will be maintained for all participants: (1) the Falls Efficacy Scale-International (FES-I, Brazilian version) to assess concern about falling; (2) the 2 min stationary march test to assess functional physical fitness; (3) bilateral surface electromyography (sEMG) of the tibialis anterior and medial gastrocnemius muscles using the EMG832C system (EMG System do Brasil, São Paulo, Brazil); and (4) posturography performed simultaneously with sEMG using a force platform (AccuSway PLUS, AMTI, Watertown, MA, USA).

### 2.2. Registry

This trial was approved by the Research Ethics Committee of the Augusto Motta University Center UNISUAM (IRB No. 7.782.752 on 21 August 2025), according to the guidelines of Law No. 14.874/24 of the National Health Council, in accordance with the Helsinki Declaration for research in humans, and prospectively registered in the Brazilian Registry of Clinical Trials (registration code: RBR-68pry5j, registration dated: 8 December 2025, WHO Universal Trial Number: U1111-1332-5737, available at https://ensaiosclinicos.gov.br/rg/RBR-68pry5j (accessed on 31 December 2025)).

### 2.3. Setting

The trial will be conducted at the Musculoskeletal Performance Laboratory of the Graduate Program in Rehabilitation Science (PPGCR/UNISUAM), Rio de Janeiro, Brazil.

### 2.4. Recruitment

The sample will be recruited intentionally and by convenience and opportunity at the Open University for Seniors (UNATI) of the Augusto Motta University Center (UNISUAM). Although participants are recruited from the UNATI/UNISUAM program, the research procedures will always be conducted by a research team independent of the regular UNATI team, as well as those responsible for routine activities.

Baseline characteristics will include sociodemographic data, history of falls, and level of physical activity. Previous falls will be recorded based on the FES-I and self-report over the past 12 months. Physical activity level will be assessed at baseline using the International Physical Activity Questionnaire (IPAQ) [20]. These variables will be described by group and considered in the interpretation of baseline comparability and potential confounding factors.

The UNATI team will not participate in randomization, allocation concealment, outcome assessment, intervention application, or statistical analysis. To minimize cross-contamination between groups, intervention sessions will be scheduled separately, and participants will be instructed not to share details of the procedures to which they were allocated with other participants during the study period. The study will be conducted at the Musculoskeletal Performance Laboratory, linked to the Postgraduate Program in Rehabilitation Sciences (PPGCR/UNISUAM), located at Rua Dona Isabel, nº 94-Bonsucesso, Rio de Janeiro-RJ, CEP 20911-300, Brazil.

### 2.5. Eligibility Criteria

Inclusion criteria will be: (1) participants aged 60 years or older; (2) participants currently enrolled in the UNATI/UNISUAM program; and (3) preserved cognitive function screened using the Mini-Mental State Examination (MMSE), adopting education-adjusted cut-off scores appropriate for the Brazilian population, in order to ensure that participants are able to understand the study procedures and complete the proposed questionnaires [21]. Participants will be excluded if they present functional limitations or medical conditions that may compromise their health or confound study outcomes; report a history of neurological disease; report any acute episode of dizziness; present abnormal fluid accumulation (edema), especially in the lower limbs; have severe visual impairment; present clinical conditions that prevent the performance of SVT, such as the presence of a cardiac pacemaker, recent lower limb surgery (within the past year), a history of deep vein thrombosis, or previous hip or knee arthroplasty; have a diagnosis of rheumatoid arthritis; use a walking assistive device; or have undergone spinal arthrodesis.

### 2.6. Randomization, Allocation, Blinding and Implementation Procedures

Participants will be randomly allocated using a computer-generated randomization sequence (https://www.random.org). The randomization system will include 192 allocations, each linked to one of three colors representing the intervention groups (blue, black, and red), corresponding to Groups A, B, and C. Allocation will be concealed using sequentially numbered, opaque, sealed envelopes, which will be opened only after completion of the initial assessments. The randomization sequence and allocation concealment procedures will be managed by a researcher not involved in outcome assessment or statistical analysis.

Participants will be informed that the study compares different intervention strategies that may provide therapeutic benefits, and that they may or may not perceive vibratory stimuli during the intervention. Due to the perceptible nature of the vibration stimulus and the absence of a sham-vibration condition, participant blinding cannot be fully guaranteed.

After the initial assessments are completed, the initial assessor (Examiner 1) will leave the testing area to remain unaware of group allocation. A licensed physiotherapist (Examiner 2), with specific training and experience in SVT, will then enter the testing area and apply the intervention according to the assigned randomized condition. Examiner 2 will remain unaware of baseline and post-intervention outcome data. After the intervention is completed, Examiner 2 will leave the room, and Examiner 1 will return to conduct the post-intervention assessments using the same standardized procedures applied during the initial assessment.

To evaluate blinding/expectancy, after completion of post-intervention assessments, participants will be asked to indicate which condition they believe they received (SVT vs. no SVT) and to rate their confidence in this judgment.

### 2.7. Criteria for Discontinuing Allocated Interventions

Adverse events will be systematically monitored before, during, and after each intervention session. Participants will be asked about symptoms such as dizziness, nausea, pain, excessive fatigue, discomfort, or any other unexpected symptoms. All adverse events will be recorded on a standardized form, including the type of event, severity, duration, need for clinical assistance, and possible relationship to the intervention. In the presence of moderate or severe adverse events, the session will be interrupted, and the participant will be referred for appropriate clinical evaluation.

### 2.8. Ancillary and Post-Trial Care

Participants who experience any type of harm related to their participation in the study will receive complementary post-trial assistance, such as necessary clinical treatment for immediate adverse effects related to trial procedures, at no cost. In addition, all participants will have guaranteed access to physiotherapy care after the trial is completed.

### 2.9. Interventions

The protocol will be performed by a trained physiotherapist who is unaware of the initial assessment and will be conducted in accordance with the requirements of the Model for Description and Replication of Interventions TIDieR [22].

In the control condition, participants will continue their routine activities offered by the UNATI/UNISUAM program and will only undergo the scheduled clinical, sociodemographic, and outcome assessments. This group will be considered a usual physical activity control group, without additional vibration stimulus or sham-vibration procedures.

Semi-squat intervention group: Participants will perform their routine activities from the UNATI/UNISUAM program and SVT in a semi-squat position (knee flexion at 120°) using the following biomechanical parameters: F = 15, 20, and 25 Hz and D = 5 mm, Apeak of 2.3 g; 4.1 g and 6.4 g. The participant will perform 1 min of vibration in each D position (D = 5 mm) with 1 min of rest between each, repeating 3 times, totaling 18 min of intervention in a total of 10 interventions, twice a week. For standardization of the D position, the base of the vibration will be marked beforehand where participants should keep their feet during the sessions. SVT will be performed at least 30 min after their activities proposed by the UNATI/UNISUAM program.

Seated intervention group: Participants will perform the routine activities of the UNATI/UNISUAM and SVT programs while seated in a chair with their hands resting on their knees. The biomechanical parameters will be the same as those reported for the semi-squat position.

Adherence to the intervention will be monitored using individual attendance logs completed at each session by the research team. The number of completed sessions, missed sessions, and reasons for absence, when available, will be recorded. Adherence will be expressed as the percentage of completed sessions relative to the total number of planned sessions. Participants who complete at least 80% of the planned sessions will be considered adherent to the intervention protocol.

**Figure 1:**
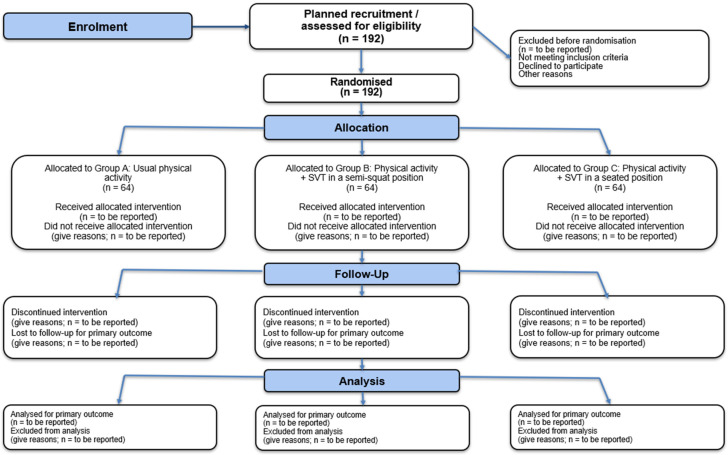
CONSORT 2025 flow diagram.

Below is a flow diagram (Figure 1) of the progress through the phases of a randomized trial of three groups (that is, enrolment, intervention allocation, follow-up, and data analysis).

### 2.10. Outcome Measures

The primary outcome will be concern about falling, assessed using the Falls Efficacy Scale-International, Brazilian version (FES-I-Brazil). Secondary outcomes will include functional physical conditioning, electromyographic activity, muscle synergism, and center of pressure oscillation.

### 2.11. Concern About Falling

Concern about falling will be assessed using the Falls Efficacy Scale-International, Brazilian version (FES-I-Brazil). This questionnaire presents questions about the perception of fear of falling when performing 16 activities. Each activity has a score from one to four, with 1 point (not at all concerned), 2 points (slightly concerned), 3 points (moderately concerned), and 4 points (extremely concerned). The total score can range from 16 to 19 points (low concern), 20 to 27 points (moderate concern), and 28 to 64 points (high concern). This instrument will be administered at baseline and within 48 h after the final intervention session [16,17,18]. In addition to analyzing FES-I as a continuous outcome within the planned Group × Time framework, interpretability will be supported by reporting the proportion of participants who shift across established concern categories (low/moderate/high) from baseline to post-intervention. Because MCID/MID thresholds for patient-reported outcomes can vary substantially across estimation methods and populations, and because credible anchor-based MIDs are not consistently available for community-dwelling older adults, the study will also apply a transparent distribution-based benchmark (0.5 baseline SD) to describe a minimal important change in FES-I as a supplementary interpretive reference rather than a substitute for the primary inferential model [23,24]. This approach is intended to strengthen clinical interpretability while avoiding overstatement of “clinical significance” in the absence of stable, population-specific anchor-based thresholds.

### 2.12. Physical Conditioning

Physical conditioning will be assessed using the 2 min stationary march test, which consists of performing the following task: the participant is instructed to perform a stationary march for 2 min, alternately raising their knees to a height marked between the midpoint of the patella and the anterior superior iliac spine (ASIS). The participant is instructed to maintain a constant pace while being stimulated by the evaluator every 30 s to perform the task, with the 2 min time being recorded. The participant may stop performing the movements if necessary and resume during the execution time, but the timer will continue running, and the evaluator will monitor and count the complete elevations of one knee (right) during the execution of the test. If the patient has any balance deficit, a support may be used. The result is given by the total count of elevations of one knee (right) for 2 min; the classification is given according to the average total number of steps according to age. In men aged 60 to 64, a performance of between 87 and 115 steps is expected; between 65 and 69 years, between 86 and 116 steps; from 70 to 74 years, between 80 and 110 steps; from 75 to 79 years, between 73 and 104 steps; from 80 to 84 years, between 71 and 103 steps; from 85 to 89 years, between 59 and 91 steps; and from 90 to 94 years, between 52 and 86 steps. For women, the reference values are slightly lower: 75 to 107 steps (60–64 years), 73 to 106 (65–69 years), 68 to 101 (70–74 years), 66 to 98 (75–79 years), 60 to 90 (80–84 years), 50 to 88 (85–89 years), and 44 to 81 (90–94 years) [25]. Interpretation of test performance is based on comparison with these normative values. Results below the expected range indicate lower functional endurance and poorer physical conditioning, possibly reflecting impairments in the cardiorespiratory system and overall mobility. On the other hand, values within or above the average for the participant’s age and sex suggest a good level of physical fitness, indicating greater functional capacity and a lower risk of physical decline.

### 2.13. Electromyography

After receiving the participant, all hairs in the area where the electrodes will be placed will be shaved. The skin must be cleaned with alcohol and abrasive paste (ANVISA Registration: 81210770020) before attaching the electromyography (sEMG) electrodes to ensure proper contact. Disposable active bipolar electrodes with silver/silver chloride contact (Ag/AgCl, Double Trace LHED4020, Shanghai Litu Medical Appliances Co., Ltd., Shanghai, China, ANVISA Registration: 8035169000) will be used, each with a diameter of 1 cm for the circular conductive area and a spacing of 2 cm between the centers. All data collection involving electromyography will follow the current recommendations proposed by [26,27].

Regarding the proposed task, the research context will be characterized by a laboratory environment, which will follow the same tasks proposed in the posturography and maximal voluntary isometric contraction section, which will be performed in a counterbalanced manner (Latin square technique) with a 2 min interval between tasks. The electrodes will be positioned on both limbs along the axes of the muscle fibers:

Medial Gastrocnemius (GM): Proximal and posterior part of the medial condyle and adjacent part of the femur, maintaining a suggestive distance of 20 mm between the electrodes. The electrodes should be positioned over most of the muscle belly (SENIAM/ISEKI) [27].

Anterior Tibial (TA): Lateral condyle and proximal half of the lateral surface of the tibia, interosseous membrane, deep fascia, and lateral intermuscular septum, maintaining a suggestive distance of 20 mm between the electrodes. The electrodes should be positioned at 1/3 of the line between the tip of the fibula and the tip of the medial malleolus (SENIAM/ISEKI) [27].

### 2.14. Posturography

For the analysis of postural control, a force platform (AccuSway PLUS, AMTI, Watertown, USA) will be used, on which participants must remain in an orthostatic position, barefoot, with their upper limbs positioned along their body. Three distinct positions will be applied: (i) participants will remain with their feet parallel, approximately hip-width apart; (ii) semi-tandem condition, in which the feet will also be parallel and hip-width apart, but with the dominant foot positioned behind the non-dominant foot, aligning the heel of the anterior foot with the plantar arch of the posterior foot; and (iii) tandem condition, in which the non-dominant foot will be positioned in front of the dominant foot. In all conditions, participants must keep their eyes open and are instructed not to move their head, arms, or torso during the task. Each condition will be performed for 60 s. Between each task, there will be a two-minute rest interval to minimize the effect of fatigue on the results. The displacement of the center of pressure (COP) will be estimated from ground reaction forces and force moments captured by the force platform. The raw data will be digitized with a sampling frequency of 50 Hz, subjected to a 5 Hz low-pass filter, and stored for later analysis. Standardization of positions aims to ensure greater consistency between measurements, minimizing variations in foot placement that could interfere with the accuracy of the results. In addition to the posturographic assessment, surface electromyographic (sEMG) signals will be collected bilaterally from the medial gastrocnemius (GM) and anterior tibial (TA) muscles to analyze muscle activation patterns and synergism between the dominant and non-dominant lower limbs, as well as the neuromuscular contribution in the different proposed conditions [27].

### 2.15. Data Management

The original data will be digitized as image files by a trained research assistant and subsequently reviewed by a second team member. All digital records will be securely stored on a password-protected computer. To ensure data integrity, a double-entry process will be implemented using automated validation procedures in a Microsoft Excel spreadsheet (Microsoft Corporation, Redmond, WA, USA), conducted remotely by an independent reviewer. Any discrepancies identified during this process will be resolved through direct comparison with the original documents.

### 2.16. Statistical Analysis

Sample size calculation

An a priori sample size calculation was performed using G*Power software (v. 3.1.9.6) [28]. A conservative medium omnibus effect size (Cohen’s f = 0.25) was adopted, consistent with methodological guidance for a priori estimation in strength and conditioning/exercise research [29]. Assuming α = 0.05, power (1 − β) = 0.80, and three parallel groups, the required total sample size for an omnibus ANOVA comparison is 159 participants (53 per group). To account for potential losses to follow-up of up to 20%, the planned recruitment target was increased to 192 participants (64 per group), ensuring adequate power for the primary analyses. Participants of both sexes, over 60 years of age (M01.060.116.100), participating in the Open University for the Elderly program at Augusto Motta University (UNATI/UNISUAM), will be included.

### 2.17. Data Analysis

Descriptive statistics will be presented as mean ± standard deviation for continuous variables and as frequency (percentage) for categorical variables. Distributional assumptions will be evaluated using Shapiro–Wilk tests and visual inspection (histograms and Q–Q plots), and homogeneity of variance will be examined (e.g., Bartlett/Levene, as appropriate). Because the trial includes three groups and two time points (baseline and post-intervention), the primary inferential objective for each outcome will be to test the Group × Time interaction, which directly evaluates whether pre–post changes differ between groups.

For outcomes meeting parametric assumptions, a mixed-design model will be applied with Group (A, B, C) as a between-subject factor and Time (baseline, post-intervention) as a within-subject factor. The main effect of Group, main effect of Time, and, critically, the Group × Time interaction will be reported. Because Time has only two levels, sphericity is not applicable. If the interaction is statistically significant, planned follow-up comparisons (simple effects) will be conducted to compare: (i) pre–post change within each group and/or (ii) between-group differences at each time point, using multiplicity-adjusted procedures (see below).

If parametric assumptions are violated, outcomes will be analyzed using an appropriate robust repeated-measures approach (e.g., transformation where suitable, or mixed-model estimation with robust standard errors), preserving the Group × Time framework. In all cases, inference will be centered on the interaction term as the test consistent with the study design.

Multiplicity control: The primary outcome (FES-I) will be tested at α = 0.05 (two-sided). Secondary outcomes will be interpreted as supportive/exploratory and will include multiplicity control to reduce false-positive risk across the outcome family. For post hoc/simple-effects contrasts following a significant interaction, *p*-values will be adjusted (e.g., Holm–Bonferroni). For multiple secondary outcomes, an additional correction strategy will be applied (e.g., false discovery rate control) to maintain interpretability across the secondary endpoint set.

Missing data: Analyses will follow an intention-to-treat principle. Missing outcome data will be described (extent, pattern, and reasons when available). The primary repeated-measures models will be implemented using estimation procedures that allow inclusion of participants with incomplete follow-up (under a missing-at-random assumption). If missingness exceeds a minimal threshold or appears differential by group, sensitivity analyses will be performed (e.g., multiple imputation and/or complete-case/per-protocol comparisons).

All analyses will be performed in SPSS (Version 21, Chicago, IL, USA), with the significance threshold set at α = 0.05.

### 2.18. Plans for Communicating Important Protocol Amendments to Relevant Parties

Important protocol modifications, such as changes to eligibility criteria, outcomes, or analyses, will be communicated to relevant parties (e.g., the Research Ethics Committee, researchers, participants, and the journal of publication).

### 2.19. Public and Patient Involvement

Public and patients were not involved in the study design. We will invite patients to participate in the development of dissemination strategies.

### 2.20. Dissemination Plans

Data will be made available upon request to the researchers responsible for the study. The results will be disseminated through presentations at a scientific congress, as well as through publication in an indexed, peer-reviewed journal.

## 3. Discussion

This study will provide information on the effects of SVT associated with a physical activity program in older adults on the risk of falls, balance, physical conditioning, and neuromuscular variables.

Developing an ideal SVT protocol in conjunction with a physical activity protocol to minimize the impacts of falls in older adults, as well as improving physical conditioning and neuromuscular variables, such as the synergism between the medial gastrocnemius and anterior tibial muscles, is important for maintaining balance and gait and preventing the risk of falls. SVT can be a safe and effective option for the elderly population. However, for this to occur, it is necessary to establish well-defined biomechanical parameters such as frequency, peak-to-peak displacement, amplitude, peak acceleration, exposure time to mechanical vibration, and rest time [12,30]. Therefore, it is important to understand and define the best existing SVT protocols and to understand if there is a difference in the individual’s positioning in the vibration platform for such conditions combined with a physical activity protocol.

Functionality will be assessed through the 2 min stationary march test [25], the perception of fall risk through the FES-I questionnaire [18], neuromuscular variables through EMG of the medial gastrocnemius and anterior tibial muscles bilaterally, in addition to the assessment of balance on the force platform in three distinct foot positions: parallel feet, semi-tandem, and tandem [31].

There is evidence that whole-body vibration interventions may improve muscle strength, balance-related outcomes, and functional mobility in older adults [32,33,34,35]. Bemben et al. [32] concluded that SVT was effective in reducing sarcopenia-related muscle strength loss in older adults. Kocic et al. [33] and Sitjà-Rabert et al. [34] reported improvements in functional mobility and balance-related outcomes in older adults after exercise-based or whole-body vibration interventions. Jepsen et al. [35], in a meta-analysis, suggested that SVT may reduce the risk of falls and fractures, but without producing an effect on bone mineral density or bone microarchitecture.

According to Meirelles et al. [36] in their systematic review, they demonstrated that there are positive effects in the use of SVT in relation to the risk of falls in the elderly, but they highlight the lack of standardization of a well-established protocol. Yang and Butler [37] concluded in a meta-analysis that SVT may benefit mobility and balance immediately, but its effect may not persist among people with stroke, suggesting that more studies with high methodological quality are needed to determine the cumulative effects of SVT in this population.

Similarly, Kocic et al. [33] added physical exercise to the usual care in nursing home residents over 65 years of age for 6 months and found a significant improvement in the time to perform on the Timed Up and Go (TUG) test, the Five Times Sit-to-Stand Test (FTSTS), and FIM scores. Furthermore, it was observed that the changes in the TUG and FTSTS test values were significantly different between the groups, as well as for the Functional Independence Measure (FIM), with better values in the EVCI group, while in the usual care group the values worsened; however, there is no evaluation of different positions during the use of SVT [34].

The comparison between semi-squatting and seated positions during the SVT is clinically relevant, since these positions may produce different neuromuscular and systemic demands [12,13,15]. In the semi-squatting position, participants are exposed to a greater axial load and greater activation of the lower limb muscles, which can increase proprioceptive stimulation and activation of the muscles involved in postural control [13,31]. In contrast, the seated position may reduce the weight-bearing demand and offer a safer and more tolerable alternative for older adults with lower functional capacity or concern about falling, since SVT may generate both local and systemic effects [14,15,20]. Furthermore, this postural strategy during SVT has been studied, but still lacks a well-defined protocol, as well as a comparison with the use of SVT in other postures in older adults [12,25,30]. Therefore, comparing these two positions can help clarify whether the therapeutic effects of SVT are mainly related to exposure to mechanical vibration alone or to the combination of vibration with active postural and muscular demands.

We expect that SVT combined with physical activity may contribute to improvements in physical fitness, mobility, and postural control, supporting the need to identify safer and more effective protocols for older adults [34,38].

This study has some limitations that should be acknowledged. First, the absence of a sham-vibration condition limits the ability to fully blind participants and does not allow complete control for nonspecific contextual effects related to device contact, time, and exposure to the laboratory environment. Second, recruitment by convenience from a single institutional program may limit the external validity of the findings. Third, although strategies will be adopted to reduce contamination between groups, participants belong to the same university-based program, which may allow exchange of information between them. Finally, the relatively short intervention period may limit the magnitude of changes in patient-centered outcomes such as concern about falling.

## Data Availability

No new data were created or analyzed in this study.
